# Improved ELISPOT protocol for monitoring Th1/Th17 T-cell response following *T*.*gondii* infection

**DOI:** 10.1371/journal.pone.0301687

**Published:** 2024-05-08

**Authors:** Francois Fasquelle, Anaïs-Camille Vreulx, Didier Betbeder

**Affiliations:** Vaxinano, Loos, France; Instituto Butantan, BRAZIL

## Abstract

In the monitoring of human *Toxoplasma gondii* infection, it is crucial to confirm the development of a specific Th1/Th17 immune response memory. The use of a simple, specific, and sensitive assay to follow the T-cell activation is thus required. Current protocols are not always specific as stimulation with peptides is Human Leukocyte Antigen (HLA)-dependent, while stimulation with total-lysis antigens tends to stimulate seronegative donors resulting to false positives. Here, an improved ELISPOT protocol is reported, using peripheral blood mononuclear cells (PBMC) of *T*.*gondii*-infected donors, incubated with the inactivated parasite. The results showed that, contrary to standard protocols, a pre-incubation step at high cell density in presence of the inactivated parasite allowed a specific Th1/Th17 response with the secretion of IFN-γ, IL-2, IL-12 and IL-17 cytokines. This protocol allows to evaluate precisely the immune response after a *T*.*gondii* infection.

## I. Introduction

*Toxoplasma gondii* is an intracellular protozoa, distributed worldwide, which infects mammals mainly via the consumption of contaminated meat or water [1]. Postnatal *T*.*gondii* infections are mostly asymptomatic and rarely require medication, as the parasite remains latent in the body in a cystic form. However, it can resurface in all the organs (mainly muscles, eyes, and brain) during sporadic episodes of immunodepression or immune suppression, leading to severe local damage. Moreover, when acquired by seronegative pregnant individuals, the parasite can cross the placenta to reach the fetus and can dramatically affect its development. Unfortunately, no drug has yet exhibited a complete satisfaction, as the classical combination of pyrimethamine and sulfadiazine induces significant side effects, and as there is still no effective treatment for the eradication of tissue cysts [2, 3].

In immunocompetent individuals, *T*.*gondii* reactivation is efficiently restrained by the establishment of a Th1 memory immune response where CD4+ and CD8+ T cells-producing IFN-γ, supported by dendritic cells and NK cells, and sustained by the secretion of co-stimulatory cytokines such as TNF-α, IL-1β, IL-2 or IL-12 [4, 5]. The secretion of IFN-γ indeed inhibits the parasite growth in infected cells, by stimulating the production of inducible nitric oxide synthase (iNOS) expression in infected macrophages, and by promoting the expression of indoleamine 2,3-dioxygense (IDO), enzyme involved in the tryptophan catabolism which is essential for the parasite survival. Moreover, cytotoxic CD8+ can kill both infected cells and extracellular parasites. Besides, the loss of a specific Th1 immunity is described to play a preponderant role in the parasite reactivation [6, 7]. Moreover, the triggering of a Th17 response has also been found to be determinant in protecting the host [8]. The monitoring of the T-cell immunity triggered by the *Toxoplasma gondii* infection is thus important [9].

T-cell based assays require the isolation of peripheral blood mononuclear cells (PBMC) followed by their stimulation with chosen antigens. The Th1/Th17 specific memory response can then be measured by different cytokine release assays, the most widely used being: (i) quantifying the percentage of intracellular cytokines in CD4^+^/CD8^+^ T-cells and their proliferation by flow cytometry; (ii) measuring the secreted cytokines by ELISA or multiplex cytokine assay; (iii) evaluating the frequency of cytokine-secreting cells by ELISPOT.

Although initially developed to reveal antibody-secreting cells, ELISPOT-based assays have become one of the most used methods to evaluate T-cell immunity, as observed recently in Covid-19 studies [10, 11]. Owing to its sensitivity, a smaller volume of blood (and thus a lower number of cells) is needed to perform the analysis and distinguish between infected and naïve donors [12]. Moreover, it is less time-consuming and cheaper than other techniques, while still being able to detect the same analytes, which can be reflective of humoral immunity (IgA, IgM and IgG subclasses), but also Th1 (IFN-γ, IL-2), Th2 (IL-4, IL-10, IL-5…) and Th17 (IL-17) immunity.

Most ELISPOT protocols stimulate PBMC with overlapping peptide pools from antigens of *T*.*gondii*. However, a large panel of antigens must first be screened because the T-cell recognition depends upon the patients’ HLA [13]. This leads consequently to an even larger panel of peptides that must be tested *in vitro* or *in silico* [14]. Moreover, the number of antigens is much greater for parasites than for virus [15].

Here we describe a simple and specific ELISPOT protocol to study the Th1/Th17 response induced after *T*.*gondii* infection, using inactivated parasites to stimulate PBMC.

## II. Material and methods

### a. Material

Histopaque-1077, Dulbecco’s phosphate buffered saline (DPBS), Roswell Park Memorial Institute medium (RPMI 1640), Fetal calf serum (FCS), Dimethylsulfoxide (DMSO), Penicillin/Streptomycin, 2-mercaptoethanol and Trypan blue 0.4% were all purchased from ThermoFisher (France). Phorbol 12-myristate 13-acetate (PMA) ionomycin calcium salt, Phytohemagglutinin-L (PHA) and Concanavalin A (ConA) were purchased from Sigma-Aldrich (France). Toxoplasma serological test (TOXOPLASMA ICT IgG-IgM) was purchased from LD Bio Diagnostic (France). ELISPOT plates were purchased from Mabtech (Sweden). MicroBCA kit was purchased from Pierce (France). Vero cells were obtained from ATCC (CCL-81), as well as *T*.*gondii* tachyzoites (RH strain, ATCC 50174), and stored in liquid nitrogen until use.

### b. Ethic statement

The study was conducted in accordance with the Declaration of Helsinki. Heparinized blood samples were spared from blood donation nonrelated to the study, of anonymous and healthy volunteers, regardless of their previous Toxoplasma infection statues, and collected by the Etablissement Français du Sang (EFS, convention PLER-UPR/2020/002). Despite they were not recruited specifically for the study, all donors gave their written consent that the blood could also be used for research purpose. Blood samples were donated from the EFS for the project with the approval and in accordance with the French Research Agency recommendations, for specific research use only. All blood samples were processed within 24h of sampling.

### c. Serology and PBMC isolation

A serology against *T*.*gondii* was performed on whole blood to determine the positive or negative infection status, by immunochromatography against anti-*T*.*gondii* IgG-IgM.

The PMBC were then isolated by Histopaque-1077 gradient. Briefly, 10 mL blood was diluted in 10 mL DPBS at room temperature. The 20-mL solution was then gently layered upon 20 mL of Histopaque-1077 and centrifuged at 400g for 30 minutes. The upper layer containing the plasma was carefully discarded, and the opaque interface containing the PBMC was transferred to a 15 mL falcon tube. The cells were then washed twice in DPBS, resuspended in freezing media (RPMI 1640 with 10% heat-inactivated FCS, 10% DMSO), and frozen at -80°C for short-term storage.

### d. Cell and parasite culture

Vero cells were cultured in supplemented RPMI 1640 (10% heat-inactivated FCS, 1% Penicillin/Streptomycin) and subcultured twice a week. *T*.*gondii* tachyzoites were stored in liquid nitrogen at 10^8^ parasites in 1 mL until use. The parasites were produced in three amplification steps:

Vero cells were seeded in T25cm^2^ flasks in 5 mL supplemented RPMI at 10^5^ cells/mL until they reach 80% confluency. Tachyzoites were thawed at 37°C, and 2x10^7^ parasites/mL were seeded into the Vero flasks. When the cells full lysis was reached, the supernatant was harvested, and the tachyzoites were counted with an hematocytometer. This process was then repeated twice in larger flasks: first by seeding 10^5^ Vero cells/mL in T75cm^2^ flasks with 10 mL supplemented RPMI, and by infecting them with 4x10^6^ parasites/mL; then by seeding 10^5^ Vero cells/mL in T225cm^2^ flasks with 30 mL supplemented RPMI, and by infecting them with 4x10^6^ parasites/mL.

Tachyzoites harvested in the largest flask were then purified from Vero cell debris by centrifugation (5 min at 600xg and at room temperature), washed in DPBS 3 times by centrifugation (7 min at 2300xg and at room temperature) and counted with an hematocytometer.

### e. Parasite inactivation

After harvesting, the parasite suspension was concentrated at 2x10^9^ parasites/mL in DPBS, and they were inactivated by 5 consecutive freeze/thaw cycles at -80°C. The full inactivation was confirmed by Vero cell infection: Vero cells were seeded in 12-well plate at 200.000 cells per well, in 2 mL of supplemented RPMI 1640. At 80% confluence, they were infected with either 2.10^8^ inactivated parasites, with 10^2^ to 10^8^ living parasites as positive control, or left uninfected as negative control. After 4 days, the Vero cells integrity was evaluated by MTT test (CellTiter 96® Non-Radioactive Cell Proliferation Assay (MTT), Promega, France) and the intracellular presence of the parasite was observed by microscopy.

Inactivated parasites were assessed for total protein content, calculated by microBCA assay.

### f. PBMC stimulation and IFN-γ ELISPOT assay

Standard protocol [16, 17]: frozen PBMC were thawed at 37°C and washed in pre-warmed, complete RPMI 1640 (10% heat inactivated FCS, 100 IU/mL penicillin, 100 mg/mL streptomycin, 50 mM 2-mercaptoethanol) by centrifugation at 400g for 10 minutes. Living cells were then counted with Trypan blue and seeded in 96-well, precoated IFN-γ ELISPOT plates (Mabtech, Sweden), in 200μL media (10^6^ cells /mL). For each donor, the cells were seeded in 3 separated wells (negative control, positive control and stimulation with the antigens). The cells were then stimulated, with either complete RPMI (negative control), with PMA/Ionomycin (50ng/mL; 1μg/mL) or PHA/ConA (5 μg/mL for both) as positive control, or with inactivated parasites (5 μg per million cells), at 37°C for 24h (Table 1). After 24h, the cells were discarded, the IFN-γ-representative spots were revealed as described by the manufacturers and counted in an Astor ELISPOT reader (Mabtech, Sweden). Antigen-specific responses were expressed as spot-forming units (SFUs) per 2x10^5^ PBMCs after subtraction of spots in negative control wells.

**Table 1:** Abstract of the standard and improved ELISPOT protocols for T-cell activation.

	Pre-incubation			ELISPOT plate		
**Protocol**	**Cell concentration**	**Inactivated parasite per 10**^**6**^ **cells**	**Time**	**Cell concentration**	**Inactivated parasite per 10**^**6**^ **cells**	**Time**
**Standard**				2x10^6^ cells/mL	0.1–5 μg	24h
**Improved**	10^7^ cells/mL	0.1–5 μg	48h	2x10^6^ cells/mL	0.1–5 μg	24h

Improved protocol: frozen PBMC were thawed at 37°C and washed in pre-warmed, complete RPMI 1640 (10% heat inactivated FCS, 100 IU/mL penicillin, 100 mg/mL streptomycin, 50 mM 2-mercaptoethanol) by centrifugation at 400g for 10 minutes. Living cells were then counted in presence of Trypan blue and seeded in 96-well plates (Fisher Scientific, France) at 10^7^ cells/mL in 100μL, for a high-density, pre-incubation time (Table 1). The cells were then stimulated, with either complete RPMI as a negative control, or inactivated parasites (0.1–5 μg per million cells) for 24 or 48h at 37°C (the stimulus for positive control was added in the next step to avoid cell mortality). Then, 2x10^5^ cells were carefully harvested and seeded in a 96-well precoated IFN-γ ELISPOT plate, in 100μL media, without washing the cells nor removing the parasite. At this step, the positive control was added in the plate in the corresponding wells, by stimulating 2x10^5^ cells in 100μL with either PMA/Ionomycin (50ng/mL; 1μg/mL) or PHA/ConA (5 μg/mL each). After 24h, the cells were discarded, the spots were revealed and counted as described above. Antigen-specific responses were expressed as spot-forming units (SFUs) per 2x10^5^ PBMCs after subtraction of spots in negative control wells. The step by step protocol is described in supporting information (S1 File).

### g. Statistical analysis

Comparison between seropositive and seronegative patients were made by two-way ANOVA followed by the Tukey test. A *p* value <0.05 was considered as statistically significant. A receiver operating characteristic (ROC) curve was plotted with Prism software based on raw data, by the Wilson/Brown method. Different ROC curves were evaluated using various cutoff and the best cutoff was defined as the one showing the greatest specificity. Data were analyzed with Prism Software (GraphPad Software Inc. 8.4.2).

## III. Results

### a. PBMC pre-incubation at high density triggers specific IFN-γ secretion

**Fig 1:**
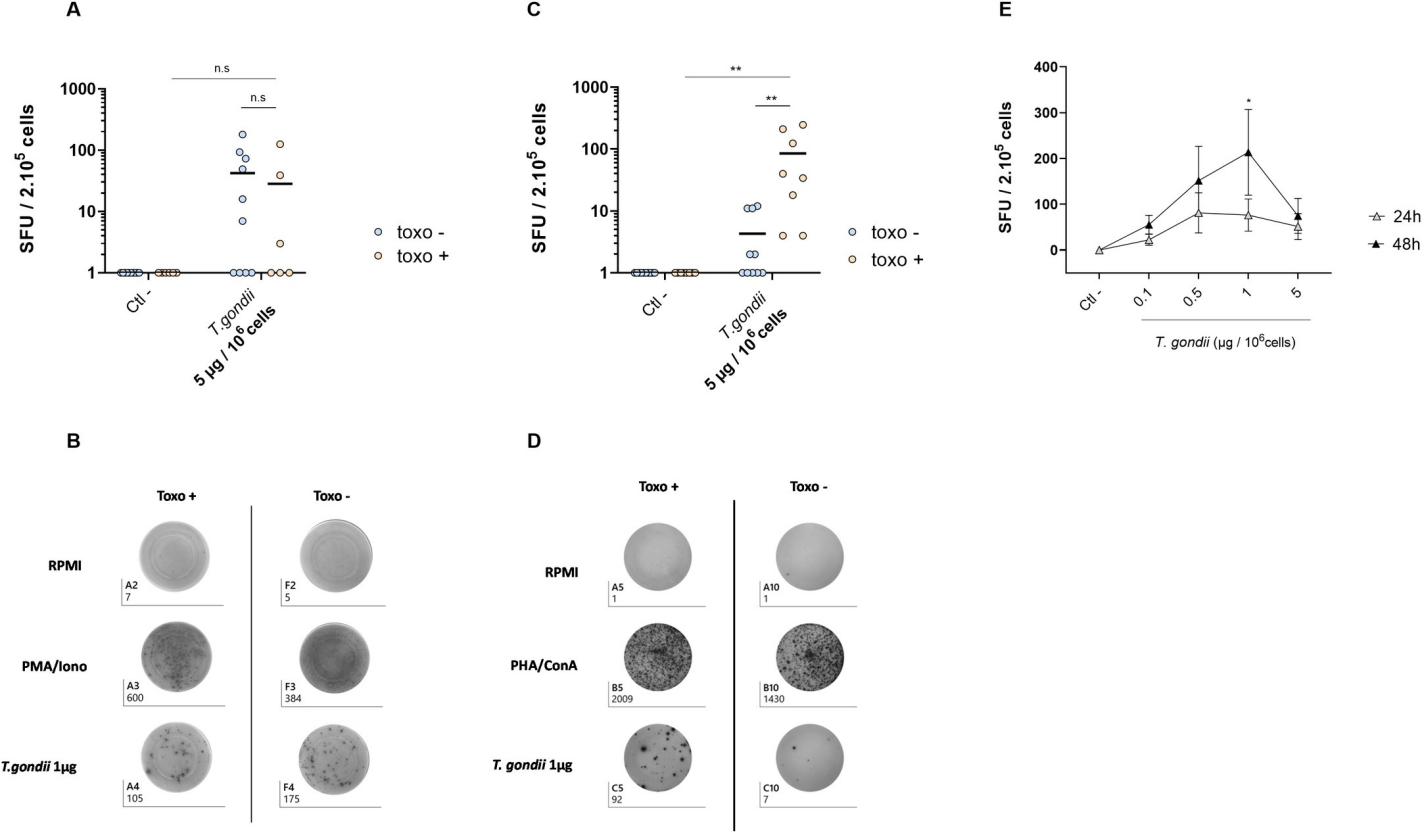
**A**. **Standard protocol**: Number of spots measured by IFN-γ ELISPOT assay from toxo+ and toxo- PBMC. **B. Standard protocol**: Representative picture of an ELISPOT plate after spot revelation. **C**. **Improved protocol**: Number of spots measured by IFN-γ ELISPOT assay from toxo+ and toxo- PBMC, adding a 24h pre-incubation at 10^7^ cells/mL with 5 μg inactivated parasite per million cells. **D**. **Improved protocol:** Representative picture of an ELISPOT plate after spot revelation. **E. Improved protocol:** Number of spots measured by IFN-γ ELISPOT assay from toxo+ PBMC, with a 24 or 48h pre-incubation at 10^7^ cells/mL with 0.1 to 5 μg inactivated parasite per million cells. * *p* <0.05, ** *p* <0.01.

PBMC were first stimulated following the standard ELISPOT protocol, at 10^6^ cells/mL and with 5 μg of inactivated parasites per million cells and for 24h. The analyses revealed IFN-γ spots regardless of the patients’ serology, with an equivalent number of spots observed between the two populations (Fig 1A and 1B). PBMC from toxo- and toxo+ blood donors were equally stimulated by the inactivated *T*.*gondii* and produced IFN-γ. This protocol failed to stimulate specifically a T-cells response on Toxoplasma-infected donors.

In order to try to improve the stimulation specificity with the inactivated parasite, PBMC were pre-cultured for 24h at a high density (10^7^ cells/mL) and in presence of 5 μg of inactivated parasites per million cells, before their incubation in the ELISPOT plate (Fig 1C and 1D). The analyses revealed a significantly higher number of IFN-γ spots for seropositive patients (mean = 85 spots) compared with seronegative patients also stimulated with the parasite (mean = 4.3 spots, *p* <0.01). This suggests that only PBMC from toxo+ blood donors were stimulated by the inactivated *T*.*gondii*. A pre-incubation step at a high cell density in presence of the inactivated parasite was able to specifically stimulate a T-cell response on toxo + blood donors, contrary to the standard protocol.

The improved conditions were fully determined by comparing 24h and 48h of pre-incubation at 10^7^ cells/mL, along with increasing quantities of inactivated parasites (0.1 μg to 5 μg of proteins per million cells). When PBMC were pre-incubated for 24h, IFN-γ spots were observed for every dose of inactivated parasites, with a maximum of 92 spots reached with 0.5 μg/10^6^ cells (Fig 1E). Furthermore, when PBMC were pre-incubated for 48h, increasing numbers of IFN-γ spots were observed for a stimulation with 0.1, 0.5 and 1 μg/10^6^ cells, leading respectively to a mean of 56, 152 and 213 (*p* <0.05) spots. However, a lower number of spots was observed when PBMC were incubated for 48h with 5 μg/10^6^ cells of antigens (mean = 75 spots). These results suggested that a 48h pre-incubation of PBMC at 10^7^ cells/mL with 1 μg of inactivated parasites per million cells are the best conditions to stimulate efficiently the memory T-cell response, and the secretion of IFN-y.

### b. Accuracy of the improved protocol

**Fig 2:**
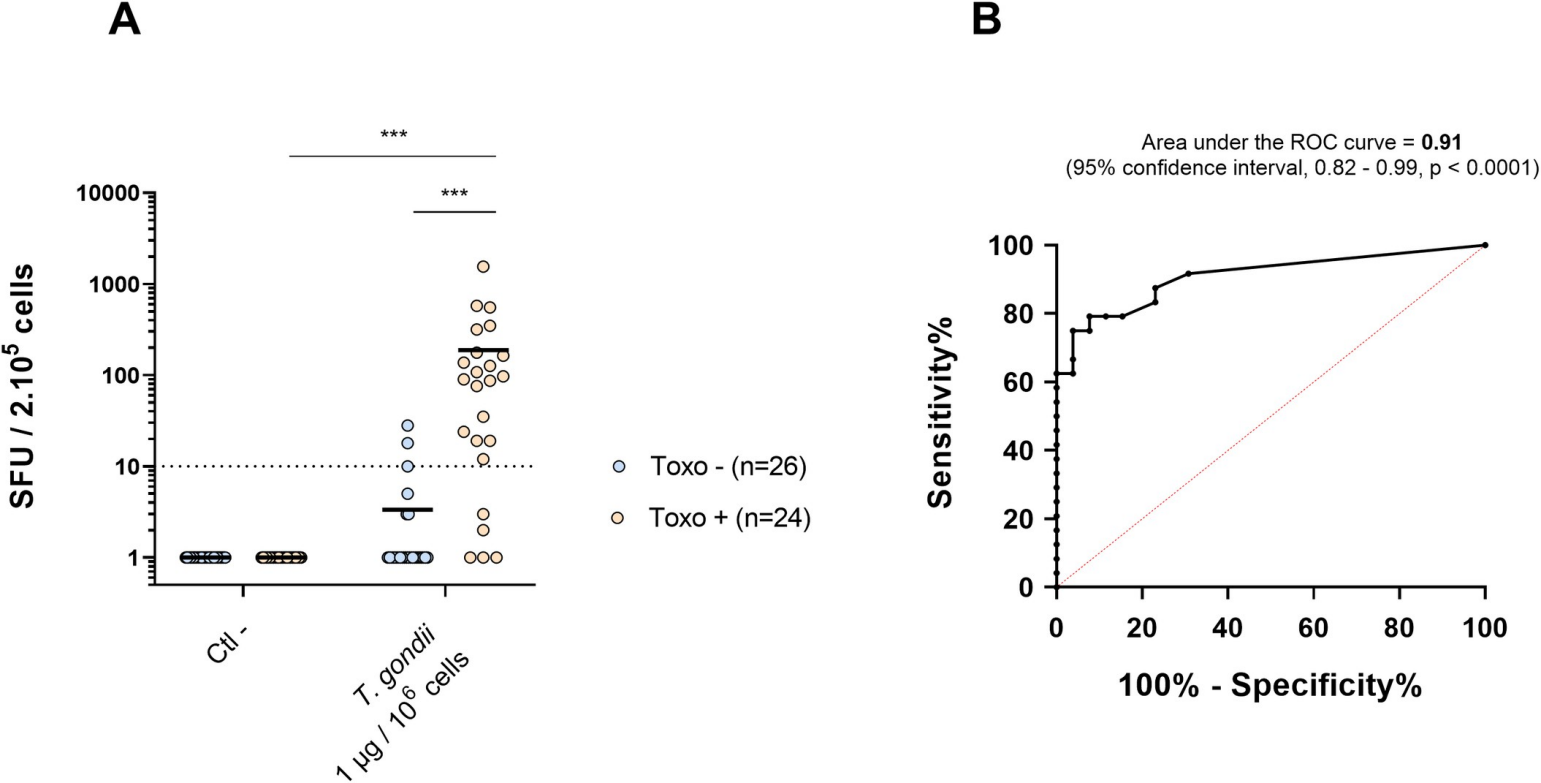
**A.** Number of spots measured by IFN-γ ELISPOT assay from toxo+ (n = 24) and toxo- (n = 26) PBMC, after a 48h pre-incubation at 10^7^ cells/mL with 1 μg of inactivated parasites per million cells. Dashed line represents the cut-off = 10 spots. **B**. ROC Curve plotted based on the ELISPOT results. *** *p* <0.001.

The sensitivity and specificity of the protocol were evaluated by stimulating PBMC from 50 patients, 26 seronegative and 24 seropositive, using the best stimulation conditions defined above: 48h pre-incubation at 10^7^ cells/mL with 1 μg of inactivated parasites per 10^6^ cells. A significantly higher number of spots was observed for PBMC from seropositive donors (mean = 188 spots) compared to seronegative subjects (mean = 3, p < 0.001), confirming the greater stimulation of PBMC from toxo+ blood donors. (Fig 2A). Furthermore, a non-specific stimulation was observed for 3/26 seronegative donors, while 19/24 seropositive donors exhibited a specific stimulation (cut-off = 10 spots), yielding a 72.2% sensitivity (95% IC, 0.63–0.95) and a 92.3% specificity (95% IC, 0.82–1.03) of the assay. The aera under the curve (AUC) of ROC curve plotted from these values was AUC = 0.91 (95% IC, 0.82–0.99) with a Youden index of J = 0.65. This confirmed the high accuracy of the assay when using this stimulation protocol (Fig 2B).

### c. PBMC pre-incubation induces a broad cellular response

**Fig 3:**
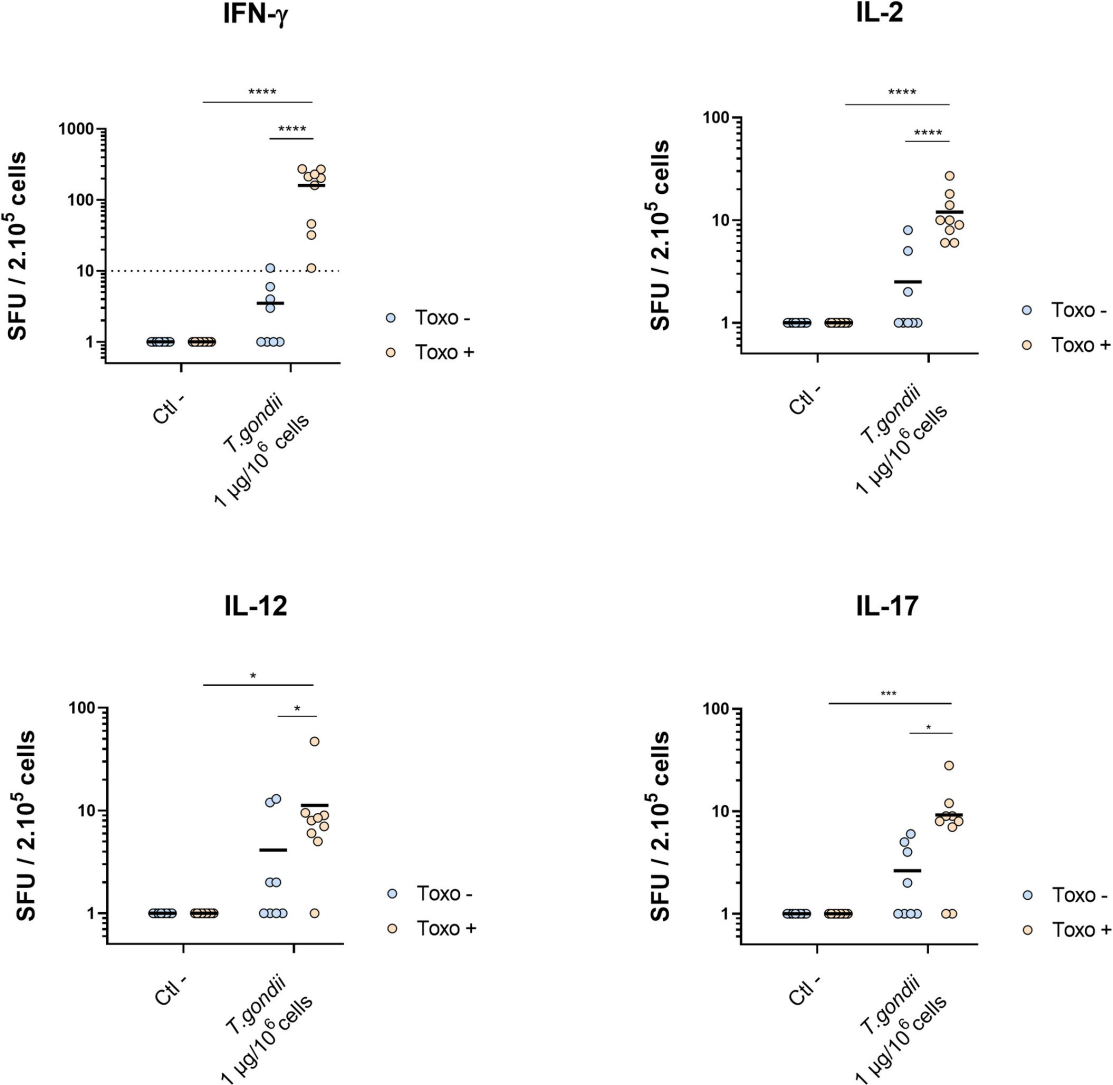
Number of spots measured by IFN-γ, IL-2, IL-12 and IL-17 ELISPOT assay from toxo+ and toxo- PBMC, after a 48h pre-incubation at 10^7^ cells/mL with 1 μg of inactivated parasites per million cells. Dashed line represents the cut-off = 10 spots. * *p* <0.05, *** *p* <0.001, **** *p* <0.0001.

The ability of this improved protocol to trigger an overall cellular immune response was assessed by measuring the secretion of IFN-γ, IL-2, IL-12 and IL-17 after stimulation (Fig 3). As observed previously, the mean number of IFN-γ spots was significantly greater for toxo+ donors compared to toxo- donors (*p* <0.0001), and the same results were observed when measuring the secretion of IL-2 (*p* <0.0001), IL-12 (*p* <0.05) and IL-17 (*p* <0.05). This suggested that a 48h pre-incubation step at a high cell density in presence of the inactivated parasite was able to trigger a specific Th1/Th17 response on PBMC from toxo + blood donors.

## IV. Discussion

The main mechanism by which T-cell activation occurs is a direct recognition of CD4^+^ and CD8^+^ lymphocyte major histocompatibility complex (MHC) by the antigens’ epitopes, thus triggering a highly specific response. In most protocols, the memory T-cell response against *T*.*gondii* is evaluated by stimulating PBMC with a peptide pool derived from the parasite’s proteins [18]. However, contrary to viral infection, a wide number of peptides should be screened here, as the recognition might be oriented to different epitopes depending on the antigen used for the immunization. For example, Huang et al. examined mouse PBMC by ELISPOT to study the reactivity of 22 peptides from 9 of the most abundant *T*.*gondii* proteins (AMA1, BAG1, GRA1/4/7, M2AP, MIC2, ROP2 and SAG1). Among them, only 5 peptides stimulated the cells of infected mice [14]. Moreover, despite being highly specific, peptide recognition is HLA dependent and the response may therefore vary among patients, and there is a significant risk of false negative responses [19, 20].

The incubation of PBMC with whole, inactivated parasites should trigger a response regardless of the antigen responsible for immunity, and regardless of the patient HLA, since this treatment contains all the parasite’s antigens in their native forms. For this reason, total lysis antigens (TLA) are often used as a positive control when evaluating the immune response of *T*.*gondii* immunized subjects. However, the greater size of the antigens compared to isolated peptides could impair MHC recognition, and unspecific immune responses often occur in seronegative people using this approach [21, 22]. Indeed, in this study too, a secretion of IFN-γ was observed for both toxo ‐ and toxo + PBMC using the standard protocol (Fig 1A), suggesting an unspecific cell activation and confirming the lack of specificity of this stimulation.

To increase the specificity of the antigen recognition, a long-term incubation at a high cell density was considered, in line with the 24-48h preincubation at a concentration above 10^6^ cells/ml often recommended, when PBMC are incubated with full-length proteins in an ELISPOT assay [23]. Antigens must indeed be first internalized, processed, and then their epitopes presented to T-cells by antigen-presenting cells (APCs) via class I/II MHC, thus triggering cytokine secretion. A higher cell concentration should indeed increase the probability of contact between APC and specific T-cells. Thus, in this study, a pre-incubation at 10^7^ cells/mL with 1 μg of inactivated parasites per million cells triggered a significantly greater IFN-γ secretion for toxo+ subjects compared with toxo- donors, confirming the higher specificity produced by this stimulation protocol (Fig 2A).

The corresponding ROC curve was plotted to evaluate the accuracy of the assay with respect to the serodiagnosis. An AUC of 0.91 was calculated (IC 95% 0.82–0.99) which is similar to or exceeds that reported for ELISPOT-based diagnostic tests [16, 17, 24] or other IFN-γ release assays (IGRA)-based diagnostic tests [25–27]. In addition, subsequent evaluation of IFN-γ, IL-2, IL-12 and IL-17 from among 17 donors showed, for the first time to our knowledge, a greater stimulation of toxo+ compared with toxo- subjects, confirming that this stimulation approach elicited a specific Th1/Th17 immune response.

## V. Conclusion

In this study, an improved ELISPOT protocol was developed to evaluate the Th1/Th17 immune response to *T*.*gondii*. It was demonstrated that a pre-incubation of PBMC at high density (10^7^ cells/mL) for 48h, in the presence of 1 μg of inactivated parasites per million cells, triggers a specific secretion of IFN-γ, IL-2, IL-12 and IL-17 among infected compared with non-infected blood donors. We suggest this more specific protocol be applied in the assessment of the T-cell memory induced by *T*.*gondii* infection.

## Supporting information

S1 File Step_by_step_ELISPOT_stimulation_protocol. (DOCX)
