# Supplementary material for: Improved ELISPOT protocol for monitoring Th1/Th17 T-cell response following T.gondii infection
Source: PLoS One. 2024 May 8;19(5):e0301687. doi: 10.1371/journal.pone.0301687 (PMC11078343; doi:10.1371/journal.pone.0301687)
Supplement: S1 File — (DOCX) [file pone.0301687.s001.docx]

**Improved ELISPOT protocol for monitoring Th1/Th17 T-cell response following *T. gondii* infection**

Francois Fasquelle^1^, Anaïs-Camille Vreulx^1^, Didier Betbeder^1^

^1^ Vaxinano, Loos, France

Abstract

In the monitoring of human *Toxoplasma gondii* infection, it is crucial to confirm the development of a specific Th1/Th17 immune response memory. The use of a simple, specific, and sensitive assay to follow the T-cell activation is thus required. Current protocols are not always specific as stimulation with peptides is Human Leukocyte Antigen (HLA)-dependent, while stimulation with total-lysis antigens tends to stimulate seronegative donors resulting to false positives. Here, an improved ELISPOT protocol is reported, using peripheral blood mononuclear cells (PBMC) of *T. gondii*-infected donors incubated with the killed parasite, to measure a specific Th1/Th17 response with the secretion of IFN-γ, IL-2, IL-12 and IL-17 cytokines.

**Keyword** : ELISPOT ; T-cell activation ; PBMC ; T. gondii

**DOI protocols.io** : dx.doi.org/10.17504/protocols.io.3byl4jp48lo5/v1

*Before Start*

This protocol involves the manipulation of human blood. Please ensure that the manipulator is vaccinated against bloodborne infections (e.g., hepatitis B…) in accordance with its country’s legislation. Please ensure that the research on human blood is allowed in your lab and that your project is validated by any reference agency or ethical comity.

*Material*

Histopaque-1077, Dulbecco's phosphate buffered saline (DPBS), Roswell Park Memorial Institute medium (RPMI 1640), Fetal calf serum (FCS), Dimethylsulfoxide (DMSO), Penicillin/Streptomycin, 2-mercaptoethanol and Trypan blue 0.4% were all purchased from ThermoFisher (France). Phytohemagglutinin-L (PHA) and Concanavalin A (ConA) were purchased from Sigma-Aldrich (France). Toxoplasma serological test (TOXOPLASMA ICT IgG-IgM) was purchased from LD Bio Diagnostic (France). MTT test (CellTiter 96® Non-Radioactive Cell Proliferation Assay (MTT)) was purchase from Promega, France). IFN-γ, IL-2, IL12 and IL-17 ELISPOT plates were purchased from Mabtech (Sweden). MicroBCA kit was purchased from Pierce (France). Vero cells were obtained from ATCC (CCL-81), as well as *T. gondii* tachyzoites (RH strain, ATCC 50174), and were stored in liquid nitrogen until use.

*Equipment*

Water bath : Isotemp GPD20, Thermofisher Scientific

Microscope for cell culture : Evos XL Core system, Invitrogen

Hematocytometer : Mallassez Blaubrand 0.2mm/0.0025mm^2^

Centrifuge : Centrifuge 5702 R, Eppendorf

Cell incubator : CellXpert C170, Eppendorf

Spectrophotometer : Multiskan Go 1510, Thermofisher Scientific

ELISPOT reader : Astor ELISPOT reader, Mabtech

*Protocol :*

- *Parasite inactivation*

1. Thaw the frozen parasite suspension in a 37°C water bath and concentrate it at 2x10^9^ parasites/mL in DPBS.
2. Inactivate the parasites by 5 consecutive freeze/thaw cycles at -80°C.
3. To confirm the total parasite inactivation, seed Vero cells 12-well plate at 200.000 cells per well, in 2 mL of supplemented RPMI 1640 (10% heat-inactivated FCS, 1% Penicillin/Streptomycin).
4. When they reach 80% confluence, infect the cells with either 2.10^8^ inactivated parasites, 10^2^ to 10^8^ living parasites as positive control, or left uninfected as negative control.
5. After 14 days, evaluate the Vero cells integrity by MTT test and observe the intracellular presence of the parasite by microscopy.
6. Assess the killed parasite total protein content by microBCA assay, according to the manufacturer instructions and using a spectrophotometer.
7. Adjust the parasite suspension at 1 mg/mL in DPBS for subsequent use and store at -80°C.

- *Serology*

1. At least 10 mL of blood from voluntary human donors are needed. Until use, keep the blood at room temperature, for less than 24h.
2. At reception, perform a serology against *T. gondii* on whole blood, with the immunochromatography test, according to the manufacturer’s instruction, to confirm the positive infection status, by against anti-*T.gondii* IgG-IgM**.**

- *PBMC isolation by Histopaque gradient*

1. Dilute 10 mL blood in 10 mL DPBS at room temperature.
2. Layer gently the 20-mL solution upon 20 mL of Histopaque-1077 at room temperature, and centrifuge at 400g for 30 minutes in a swing-rotor centrifuge. Verify the brake and acceleration are on lowest setting on centrifuge, as sharp braking and acceleration will damage the layer separation.
3. Transfer the opaque interface containing the PBMC (approx. 5 mL) to a 15 mL falcon tube.
4. Add gently 5 mL DPBS upon the cell suspension, and centrifuge at 400g for 10 minutes.
5. Discard the supernatant and wash as step 10. again.
6. Discard the supernatant, resuspend the cells in 1mL freezing media (RPMI 1640 with 10% heat-inactivated FCS, 10% DMSO), and store them at -80°C for short-term (up to 6 months) storage.

- *PBMC stimulation and IFN-γ ELISPOT assay*

1. Prepare complemented RPMI 1640, adding 10% heat inactivated FCS, 100 IU/mL penicillin, 100 mg/mL streptomycin, 50 mM 2-mercaptoethanol, and heat it at 37°C.
2. Thaw the frozen PBMC in a 37°C water bath.
3. Transfer gently the 1 mL cell suspension in 10 mL of warm complemented RPMI 1640.
4. Wash the cells by centrifugation at 400g for 10 minutes.
5. Discard the supernatant and resuspend the cells in 1 mL complemented RPMI.
6. Count living cells with Trypan blue 0.4% 1:1 (v/v), with an hematocytometer.
7. Seed PBMC in 96-well plates (Fisher Scientific, France) at 10^7^ cells/mL in complemented RPMI, in 100 µL final volume (10^6^ cells per well). Prepare 3 wells per patient : for the negative control, for the positive control and for the stimulation with the antigens.
8. Add the different stimulations : 1 µL RPMI as a negative control, or 1 µL of the 1 mg/mL killed parasites solution (1 µg parasite per million cells). The stimulus for the positive control is added in a next step.
9. Incubate the plate for 48 hours at 37°C.
10. Then, for each condition, harvest carefully 2x10^5^ cells (20 µL) and seed them in 96-well precoated ELISPOT plates, in complemented RPMI and in 100µL final volume. The cells are not washed before transferring them in the ELISPOT plate.
11. Add 1 µL of PHA and ConA (5 µg/mL each) as positive control in the corresponding wells.
12. Incubate the ELISPOT plates for 24 hours at 37°C.
13. Discarded the cells and reveal the spots following the manufacturer’s instructions.
14. Count the number of spots in an automatic Astor ELISPOT reader. Antigen-specific responses are expressed as spot-forming units (SFUs) per 2x10^5^ PBMCs.

*Additional notes*

1. Positive control needs to be added only when the cells are seeded in the 96-well ELISPOT plate.
2. The cells should not be washed when transferring in the ELISPOT plate.
3. When discarding the cells from the ELISPOT plate and performing the plate washing, be careful not to touch the bottom of the wells with the pipette, as it would produce scratches that could be counted as spots by the ELISPOT reader.
4. Antigen-specific responses can be expressed as spot-forming units (SFUs) per 2x10^5^ PBMCs or normalized to 10^6^ cells for easiest comparison with the literature.
5. The number of spots is usually 10 times higher for IFN-γ compared to the three other cytokines. Increase the number of cells in the ELISPOT plate from 2.10^5^ to 3.10^5^ cells per well could help increase the detection of rare events.
